# Longitudinal Metabolic Risk Factors Are Associated With Microvascular Complications

**DOI:** 10.1111/dom.71338

**Published:** 2026-09-13

**Authors:** Cody H. Makinson, Jihyun Park, Eva L. Feldman, Brian C. Callaghan, Evan L. Reynolds

**Affiliations:** ^1^ Department of Epidemiology and Biostatistics Michigan State University East Lansing Michigan USA; ^2^ Department of Neurology University of Michigan Ann Arbor Michigan USA

**Keywords:** chronic kidney disease, longitudinal metabolic summaries, metabolic syndrome, peripheral neuropathy, retinopathy

## Abstract

**Objective:**

Determine associations between summaries of longitudinal metabolic risk factors and microvascular complications.

**Methods:**

We performed a secondary analysis of participants with and without diabetes enrolled within the Atherosclerosis Risk in Communities Study that completed metabolic risk factors assessments four times across 11 years. We calculated slope and area under the curve (AUC) as summaries of longitudinal data for fasting glucose, systolic blood pressure (SBP), high‐density lipoprotein, triglycerides and waist circumference. We assessed Chronic Kidney Disease (CKD) with estimated Glomerular Filtration Rate, Peripheral Neuropathy (PN) with monofilament testing and Retinopathy (RN) with the Early Treatment Diabetic Retinopathy Study scale. Area under the receiver operating characteristic curve (AUROC) determined discriminatory capability of models based on slope and AUC compared to models based on metabolic risk factors at baseline.

**Results:**

We identified 4204, 2788 and 1792 persons with assessments of CKD, PN and RN, respectively. The mean age was 52.3, 57.1% were female, and 21.0% had diabetes. Logistic regression revealed fasting glucose AUC (1.09, 1.05–1.15) and SBP AUC (1.27, 1.19–1.36) associated with CKD, triglyceride AUC (1.14, 1.04–1.25) and waist AUC (1.11, 1.07–1.15) associated with PN and fasting glucose AUC (1.13, 1.04–1.22) associated with RN. Longitudinal metabolic summaries only improved AUROC by 0.001–0.01 versus models with cross‐sectional metabolic assessments.

**Conclusion:**

AUCs of longitudinal metabolic risk factors, but not slopes, were associated with microvascular complications. This finding highlights the importance that sustained metabolic impairment has for the development of microvascular complications. Surprisingly, however, in this cohort, summaries of longitudinal metabolic risk factors did not meaningfully add discriminatory capability beyond models based on metabolic assessments at baseline.

## Research in Context

- What is already known about this subject?
  ○ Although diabetes is the primary driver of microvascular complications, past studies found that other individual Metabolic Syndrome risk factors also increase the risk of microvascular complications.
  ○ Metabolic risk factors change substantially across the lifespan; however, it is unknown whether these rates of change and/or cumulative effects of metabolic risk factors are associated with microvascular complications.
  ○ Past studies largely focus either on establishing associations between microvascular complications and cross‐sectional profiles of multiple metabolic risk factors or focus only on a single longitudinal metabolic risk factor.
- What is the key question?
  ○ Are summaries of longitudinal metabolic risk factors associated with chronic kidney disease, peripheral neuropathy and retinopathy?
- What are the new findings?
  ○ We found that larger areas under the curve of longitudinal metabolic profiles were associated with each complication, and the most significant summaries varied in persons based on participant age.
  ○ Despite this, models with slopes and AUCs as exposures did not improve our ability to estimate risk of developing complications compared to models based on metabolic risk factor assessments taken at a single timepoint.
- How might this impact on clinical practice in the foreseeable future?
  ○ These findings highlight the importance that sustained metabolic impairment, but not necessarily the rate of change of metabolic impairment, has for future microvascular complications.
  ○ In addition, our findings indicate that longitudinal summaries of metabolic risk factors did not add clinically meaningful predictive abilities beyond a baseline cross‐sectional assessment.

## 1 Introduction

As of 2025, it is estimated that 589 million adults aged 20–79 years worldwide have diabetes, and it is projected that 853 million adults will be living with diabetes by 2050 [1]. Diabetes results in multiple morbid microvascular complications, including chronic kidney disease (CKD), peripheral neuropathy (PN) and retinopathy (RN). Although diabetes is the primary driver of microvascular complications, meta‐analyses have also found that other individual Metabolic Syndrome risk factors such as elevated systolic blood pressure (SBP), low high‐density lipoprotein (HDL) cholesterol, elevated triglycerides and increased waist circumference, also increase the risk of microvascular complications [2, 3, 4].

Select studies have identified associations between CKD and longitudinal Metabolic Syndrome risk factors, finding that elevated or increasing longitudinal hyperglycaemia [5, 6, 7], hypertension [8, 9] and obesity [10, 11, 12, 13] are associated with higher risk of developing CKD. One notable study of 24 094 South Korean adults aged 40–79, which simultaneously assessed multiple metabolic risk factors, found higher longitudinal assessments of SBP and triglycerides, but not obesity or total cholesterol, were associated with CKD decline [9]. In contrast, fewer studies have assessed the associations between longitudinal metabolic risk factors and either PN or RN, and these studies have typically focused on hyperglycaemia. For PN, the most robust study to date included 452 Danish adults and reported that rapid increases in haemoglobin A1c (HbA1c), but not other metabolic risk factors associated with PN [14]. In contrast, an analysis of youth with diabetes found area under the HbA1c curve was not associated with PN [15]. For RN, studies of United States youth with diabetes (*n* = 457) [16], United States adults (*n* = 2066) [17], Finnish adults (*n* = 505) [18], Taiwanese adults (*n* = 1091) [19] and Chinese adults (*n* = 3645) [20] have found that greater area under the HbA1c curves or faster increases of HbA1c are associated with RN.

However, most longitudinal studies examining metabolic risk factors and microvascular complications have evaluated single metabolic risk factors in isolation and have been limited by relatively short follow‐up durations (median ~ 9.25 years). Additionally, these studies have typically related microvascular complications to broad trajectory categories such as ‘stable and elevated’, ‘increasing’, or ‘decreasing’, rather than to more granular longitudinal patterns. Finally, no study has determined the age where longitudinal metabolic risk factors have the strongest association with future microvascular complications.

To address these limitations, in the present study we determined associations between longitudinal metabolic risk factors and future microvascular complications. Specifically, we calculated the slope and areas under the curve to summarise the rate of change and cumulative effect of longitudinal metabolic risk factors. In addition, we compared predictive performance of models developed using summaries of longitudinal metabolic risk factors to those developed using metabolic risk factor assessments at a single timepoint. Finally, we determined the ages where longitudinal metabolic risk factors were most associated with future microvascular complications.

## 2 Methods

### 2.1 Population, Study Design and Inclusion Criteria

We performed a secondary analysis of the longitudinal Atherosclerosis Risk in Communities Study (ARIC). ARIC enrolled 15 792 Black and White participants between the ages of 44 and 65 in 1987 from four sites including: Washington County, MD, Forsyth County, NC, Jackson, MS and Minneapolis, MN. After enrollment, participants completed follow‐up visits every 3 years. We required participants to have complete assessments of all metabolic risk factors at visit 1 (1987–1989), visit 2 (1990–1992), visit 3 (1993–1995) and visit 4 (1996–1998). Of those with complete metabolic phenotyping, we developed three separate analytic groups: those that completed assessments of CKD (visit 5, 2011–2013), PN (visit 6, 2016–2017) and RN (visit 5, 2011–2013). We included participants with and without diabetes.

### 2.2 Metabolic Risk Factors

In each follow‐up visit, participants underwent assessments of 8‐h fasting glucose (unit = mg/dL), SBP (unit = mmHg), HDL (unit = mg/dL), triglycerides (unit = mg/dL) and waist circumference (unit = cm). HbA1c (visits 4–6) and glucose tolerance testing (visit 4) were also collected during follow‐up. Diabetes was defined throughout the 4 visits according to the 2025 American Diabetes Association Standards of Care, as having an 8‐h fasting glucose ≥ 126 mg/dL, 2‐h glucose tolerance testing level ≥ 200 mg/dL, HbA1c ≥ 6.5% (48 mmol/mol) or if participants self‐reported having diabetes or taking diabetes medicine at visit 1 [21]. If any of the criteria for diabetes were met at any visit 1–4, a person was considered as having diabetes.

### 2.3 Longitudinal Summaries of Metabolic Risk Factor From Visits 1 to 4

We calculated the slope and area under the curve (AUC) from visit 1 to visit 4 to summarise the longitudinal data of each metabolic risk factor. Slopes were calculated using ordinary least squares regression for each participant's measurements from visits 1–4 as a function of follow‐up time. AUC for metabolic risk factors during follow‐up was calculated using the trapezoid rule [22]. We also determined cross‐sectional metabolic risk factors at visit 1 (1987–1989).

All slopes are measured per 1‐year increase. AUC of fasting glucose, SBP, and HDL were all scaled to represent 10‐unit (mg/dL, mmHg, mg/dL, respectively), triglycerides a 50 mg/dL, and waist circumference a 5 cm change.

### 2.4 Microvascular Complications at Visits 5 and 6

CKD was defined as having estimated Glomerular Filtration Rate (eGFR) < 60 mL/min/1.73 m^2^ at visit 5 using the 2021 CKD‐EPI Creatinine equation [23]. PN was assessed using Semmes‐Weinstein 10‐g monofilament testing at three sites on each foot and defined as having at least one insensate site at visit 6 [24, 25]. RN was defined as having at least first‐stage (mild) proliferative diabetic retinopathy via an Early Treatment Diabetic Retinopathy Study (ETDRS) score ≥ 61 at visit 5 [26, 27, 28]. Participants that had eGFR < 60 at any visits 1–4 were excluded from the CKD cohort, and those that self‐reported having RN at visit 3 were excluded from the RN cohort.

### 2.5 Statistical Analysis

Our primary analysis was to establish associations between summaries of longitudinal metabolic risk factors and future microvascular complications. Our exploratory analysis assessed whether the association between longitudinal metabolic risk factors and complications changes with age. Given the number of comparisons in our primary analysis (30 parameters of interest in longitudinal summaries, 15 parameters of interest in cross‐sectional models), Benjamini–Hochberg (B–H) corrected *p* values were evaluated to determine statistical significance for our primary analysis, and sensitivity analyses at the significance level of 0.05 (B–H adjusted).

Descriptive statistics were calculated to compare the demographic information and metabolic risk factors between the analysis cohorts and the entire ARIC cohort, and to compare those with vs. without each microvascular complication. Independent two‐sample *t*‐tests were used to determine differences in metabolic risk factors between participants with compared to those without each microvascular complication.

Our primary analysis fit logistic regression models to determine the association between the slope and AUC for all five longitudinal metabolic risk factors from visits 1 to 4 with CKD (visit 5), PN (visit 6) and RN (visit 5) at visits 5 or 6. Each model adjusted for age, race, sex and height. The model for CKD also adjusted for eGFR at visit 4. Wald tests were performed to make inference on the effects of longitudinal metabolic summaries on microvascular complication outcomes, and variance inflation factors were assessed to ensure the models were free of multicollinearity. To compare our longitudinal models to cross‐sectional models, we also fit multivariable logistic regression models to determine the association between cross‐sectional metabolic risk factors assessed at visit 1 and visit 4, with odds of CKD, PN and RN at visits 5 or 6.

To evaluate discriminatory capability, we calculated the area under the Receiver Operator Characteristic Curves (AUROC), sensitivity, specificity, negative predictive value (NPV) and positive predictive value (PPV) for each model. We confirmed our models were well‐calibrated by evaluating calibration plots and performing Hosmer–Lemeshow tests. To evaluate prediction accuracy, we calculated Brier scores. DeLong's test, Integrated Discrimination Improvement (IDI) and Net Reclassification Improvement (NRI) were calculated to determine whether inclusion of longitudinal metabolic risk factor summaries from visits 1 to 4 improved discriminative and predictive capabilities compared to cross‐sectional models at visit 1 and visit 4. Finally, we included a decision curve analysis to visualise the impact of our models on hypothetical clinical decision making.

We performed four sensitivity analyses of our primary analysis. First, to determine whether the associations between metabolic risk factors and microvascular complications differed based on diabetes status, we re‐fit the above models for participants that developed diabetes by visit 4 and also those who did not. In our second sensitivity analysis, given that AUCs may be highly correlated to baseline metabolic risk factors, we re‐fit our primary analysis, including the baseline and slope of each metabolic risk factor for CKD, PN and RN, instead of slopes and AUCs. Third, to compare whether results were consistent when including participants that had incomplete follow‐up, we performed multiple imputations with chained equations and re‐fit our primary regression models including all study participants. Specifically, we used Predictive Mean Matching as the imputation method, creating 50 imputed datasets, each with 25 iterations. We then used Rubin's Rule to pool the results and obtain odds ratios and 95% confidence intervals for slopes and AUCs of longitudinal metabolic risk factors. Finally, to determine the consistency of the associations between summaries of longitudinal metabolic risk factors and microvascular complications upon adjusting for additional covariates, we re‐fit our primary regression models, also including possible confounding factors. In particular, we re‐fit our primary models, also adjusting for smoking status, alcohol intake, educational attainment, depression measured using the 11‐item Center for Epidemiologic Studies Depression (CES‐D) scale, social isolation assessed with the Lubben Social Network Scale (LSNS), household income, physical activity assessed with the Baecke questionnaire, cholesterol and hypertension medication use and duration of diabetes.

In our exploratory analysis, to determine whether changes to metabolic risk factors at different age periods (44–49, 50–54, 55–59, 60–65) have differential associations with CKD, PN and RN. As a secondary analysis, we re‐fit our primary logistic models, including interaction effects between age groups and the longitudinal slopes and AUCs of each metabolic risk factor to assess the significance of interaction effects and marginal associations.

Complete case analysis was used to handle missing data. We assumed that data are missing at random for all analysis. All analyses were completed using R Studio version 4.4.2.

## 3 Results

### 3.1 Study Participation, Demographic Information and Metabolic Risk Factors

Of the 15 792 total participants, 8433 (53.4%) had complete data for age, race, sex, height, fasting glucose, SBP, HDL, triglycerides and waist circumference for visits 1–4. Among those with complete demographic and metabolic data, 4475 (28.3%) had eGFR assessed at visits 1–5, 2788 (17.7%) had monofilament testing at visit 6 and 1914 (12.1%) had ETDRS assessed at visit 5. We identified and excluded 271 participants with CKD at visits 1–4 and 122 with RN at visit 3 from the respective analyses. The final models consisted of 4204 (26.6%) in the CKD cohort, 2788 (17.7%) in the PN cohort and 1792 (11.3%) in the RN cohort (Figure 1).

**FIGURE 1:**
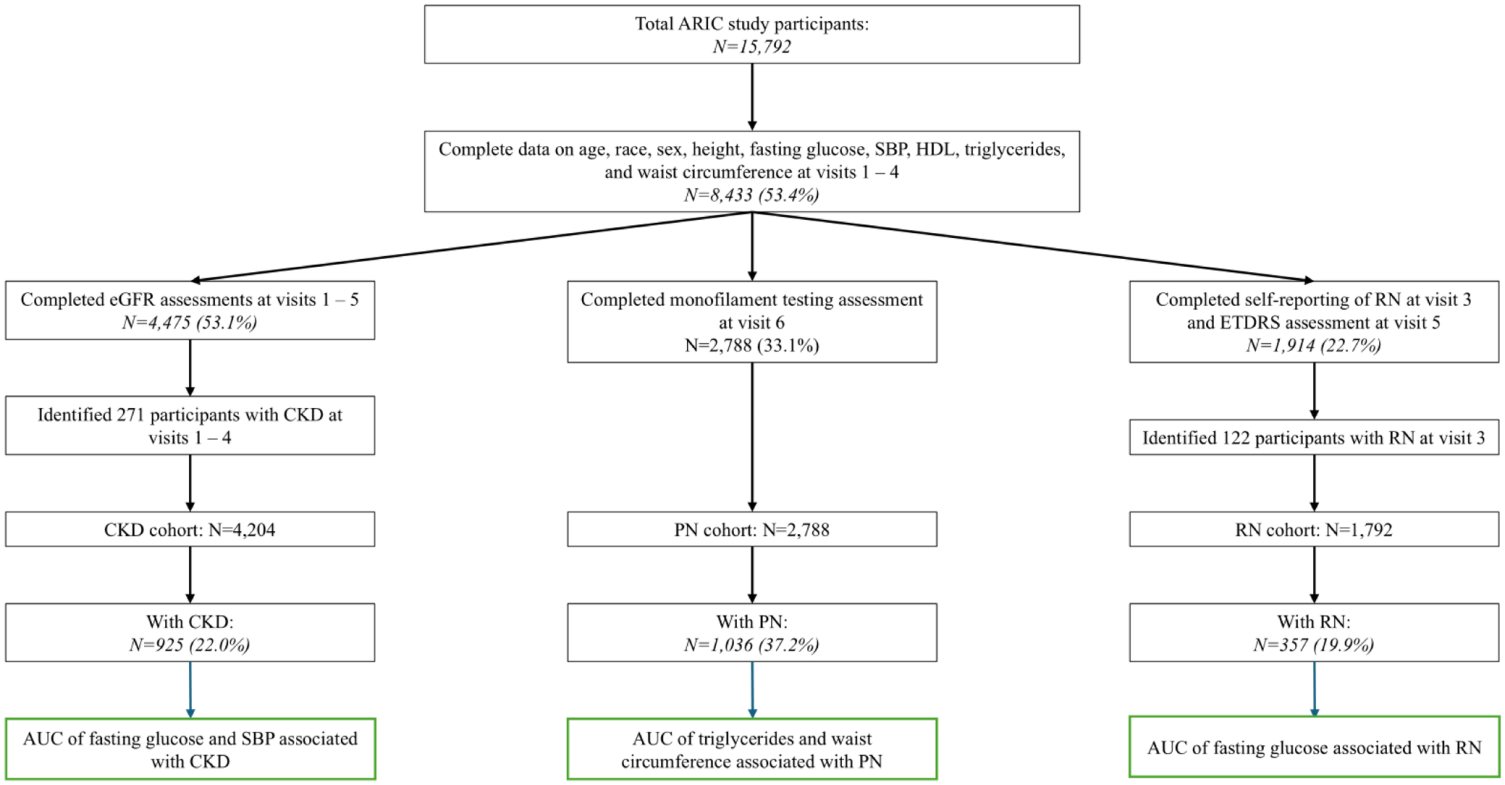
Inclusion criteria for CKD, PN and RN cohorts. ARIC: atherosclerosis Risk in Communities Study; AUC: area under curve; CKD: chronic kidney disease; eGFR: estimated glomerular filtration rate; ETDRS: early treatment diabetic retinopathy study; PN: peripheral neuropathy; RN: retinopathy; SBP: systolic blood pressure, HDL: high‐density lipoproteins.

In the CKD cohort, at visit 1, the mean (standard deviation [SD]) age was 52.2 (5.1) years, 14.2% were Black, 85.8% were White, and 57.4% were female. At visit 1, fasting glucose was 99.5 mg/dL (17.5), SBP was 136.4 mmHg (14.0), HDL was 53.2 mg/dL (16.3), triglycerides was 115.5 mg/dL (57.7) and waist circumference was 94.3 cm (12.7) (Table 1). The PN and RN cohorts had similar demographic and metabolic profiles to the CKD cohort. The proportion of participants with diabetes increased from 3.8% at visit 1 to 21.8% at visit 4 in the CKD cohort, from 2.7% to 18.0% in the PN cohort and from 3.5% to 23.2% in the RN cohort. We found that 22.0% had CKD at visit 5, 37.2% had PN at visit 6, and 19.9% had RN at visit 5. Of the 4540 subjects, 56.3% did not develop any of the microvascular complications, 36.6% developed one, 6.7% developed two, and 0.4% developed all three complications. Compared to the analysis cohorts, the full ARIC cohort had larger fasting glucose (mean (SD): 107.0 (34.2)) and triglycerides (131.3 (80.8)) (Table 1). In addition, the full ARIC cohort were older (54.3 (5.8)) and included more Black participants (3576 (24.6%)).

**TABLE 1:** Demographic and metabolic profiles of participants overall, and stratified by presence of CKD, PN and RN.

Variables	Overall cohort	Total CKD cohort	CKD	No CKD	Total PN cohort	PN	No PN	Total RN cohort	RN	No RN
Sample size										
Total	15 277	4204	925	3279	2788	1036	1752	1792	357	1435
44–49	3803	1543	217	1326	1215	354	861	474	62	412
50–54	3741	1296	254	1042	883	346	537	539	93	446
55–59	3580	926	275	651	512	235	277	521	127	394
60–65	3404	439	179	260	178	101	77	258	75	183
Demographic										
Diabetes		*			*			*		
Yes		918 (21.8%)	267 (28.9%)	651 (19.9%)	502 (18.0%)	213 (20.6%)	289 (16.5%)	415 (23.2%)	100 (28.0%)	315 (22.0%)
Visit 1 age	54.3 (5.8)	52.2 (5.1)*	54.2 (5.4)	51.6 (4.9)	51.2 (4.7)*	52.3 (4.9)	50.5 (4.5)	53.4 (5.2)*	55.0 (5.1)	53.0 (5.2)
Race		*			*			*		
Black	3576 (24.6%)	599 (14.2%)	176 (19.0%)	423 (12.9%)	444 (15.9%)	154 (14.9%)	290 (16.6%)	268 (15.0%)	79 (22.1%)	189 (13.2%)
White	10 952 (75.4%)	3605 (85.8%)	749 (81.0%)	2856 (87.1%)	2344 (84.1%)	882 (85.1%)	1462 (83.4%)	1524 (85.0%)	278 (77.9%)	1246 (86.8%)
Sex		*			*			*		
Female	7883 (54.3%)	2413 (57.4%)	519 (56.1%)	1894 (57.8%)	1628 (58.4%)	455 (43.9%)	1173 (67.0%)	997 (55.6%)	166 (46.5%)	831 (57.9%)
Metabolic										
Visit 1 fasting glucose (mg/dL)	107.0 (34.2)	99.5 (17.5)*	102.7 (25.0)	98.6 (14.6)	98.4 (15.0)*	100.1 (14.3)	97.4 (15.3)	99.0 (15.1)*	101.9 (21.4)	98.3 (13.0)
Fasting glucose slope (mg/dL)		0.3 (2.3)*	0.6 (3.1)	0.3 (2.0)	0.3 (2.0)	0.3 (2.1)	0.3 (2.0)	0.4 (2.3)	0.5 (2.7)	0.4 (2.1)
Fasting glucose AUC (10 mg/dL)		102.5 (18.2)*	106.5 (23.7)	101.3 (16.1)	101.3 (16.1)*	103.3 (17.2)	100.1 (15.4)	102.3 (15.7)*	105.7 (19.9)	101.4 (14.4)
Visit 1 SBP (mmHg)	141.5 (17.3)	136.4 (14.0)*	140.7 (15.0)	135.2 (13.5)	135.2 (13.6)*	136.4 (13.7)	134.5 (13.6)	136.6 (14.1)*	140.2 (15.6)	135.7 (13.6)
SBP slope (mmHg)		1.4 (1.7)*	1.6 (1.9)	1.3 (1.7)	1.4 (1.7)	1.3 (1.7)	1.4 (1.6)	1.4 (1.7)	1.5 (1.9)	1.4 (1.7)
SBP AUC (10 mmHg)		142.7 (13.3)*	147.7 (13.9)	141.3 (12.8)	141.6 (12.9) *	142.8 (12.9)	140.8 (12.8)	143.0 (13.1)*	146.1 (13.8)	142.2 (12.9)
Visit 1 HDL (mg/dL)	51.4 (16.9)	53.2 (16.3)*	51.8 (16.5)	53.6 (16.2)	53.7 (16.2)*	51.1 (15.9)	55.2 (16.3)	53.1 (16.4)	52.1 (16.3)	53.3 (16.4)
HDL slope (mg/dL)		−0.1 (1.2)	−0.1 (1.1)	−0.1 (1.2)	−0.1 (1.2)	−0.1 (1.1)	−0.1 (1.2)	−0.2 (1.1)	−0.2 (1.1)	−0.2 (1.2)
HDL AUC (10 mg/dL)		52.3 (15.6)*	50.8 (15.2)	52.8 (15.7)	52.8 (15.6)*	50.1 (15.0)	54.4 (15.7)	51.9 (15.5)	50.9 (15.4)	52.2 (15.6)
Visit 1 triglycerides (mg/dL)	131.3 (80.8)	115.5 (57.7)*	125.9 (63.6)	112.5 (55.6)	112.4 (56.3)*	121.5 (61.4)	107.1 (52.3)	116.7 (56.5)	121.3 (58.9)	115.6 (55.8)
Triglycerides slope (mg/dL)		2.3 (6.3)*	1.7 (6.5)	2.5 (6.2)	2.4 (6.3)*	1.7 (6.5)	2.8 (6.2)	2.3 (6.1)*	1.7 (6.3)	2.5 (6.0)
Triglycerides AUC (50 mg/dL)		126.2 (52.7)*	133.6 (55.8)	124.2 (51.6)	123.1 (51.1) *	129.2 (54.1)	119.6 (48.9)	127.7 (52.4)	129.0 (52.3)	127.4 (52.4)
Visit 1 waist circumference (cm)	97.1 (13.9)	94.3 (12.7)*	96.8 (12.4)	93.7 (12.7)	93.7 (12.6)*	97.1 (12.4)	91.7 (12.3)	94.8 (12.5)*	96.1 (11.7)	94.4 (12.7)
Waist circumference slope (cm)		0.7 (0.8)	0.7 (0.8)	0.7 (0.8)	0.8 (0.8)*	0.7 (0.8)	0.8 (0.8)	0.7 (0.8)*	0.6 (0.7)	0.8 (0.8)
Waist circumference AUC (5 cm)		97.3 (12.8)*	99.7 (12.7)	96.6 (12.8)	96.8 (12.7) *	100.0 (12.5)	94.9 (12.5)	97.7 (12.8)	98.7 (12.1)	97.5 (12.9)

*Note: p* values: *: < 0.05.

Abbreviations: AUC: area under the curve; CKD: chronic kidney disease; HDL: high‐density lipoprotein; PN: peripheral neuropathy; RN: retinopathy; SBP: systolic blood pressure.

### 3.2 Unadjusted Association of Longitudinal Metabolic Risk Factor Summaries (From Visits 1 to 4) and Microvascular Complications (at Visit 5 or 6)

Participants with CKD had larger slopes of fasting glucose (0.6 [3.1] vs. 0.3 [2.0]) (*p* = 0.007) and SBP (1.6 [1.9] vs. 1.3 [1.7]) (*p* < 0.001), but a smaller slope of triglycerides (1.7 [6.5] vs. 2.5 [6.2]) (*p* < 0.001) compared to those without CKD (Table 1). HDL and waist circumference slopes were not different between those with and without CKD. Those with CKD had larger AUCs of fasting glucose (106.5 [23.7] vs. 101.3 [16.1]) (*p* < 0.001), SBP (147.7 [13.9] vs. 141.3 [12.8]) (*p* < 0.001), triglycerides (133.6 [55.8] vs. 124.2 [51.6]) (*p* < 0.001), waist circumference (99.7 [12.7] vs. 96.6 [12.8]) (*p* < 0.001), and a smaller AUC of HDL (50.8 [15.2] vs. 52.8 [15.7]) (*p* < 0.001) compared to those without CKD.

Those with PN had smaller slopes of triglycerides (1.7 [6.5] vs. 2.8 [6.2]) (*p* < 0.001) and waist circumference (0.7 [0.8] vs. 0.8 [0.8]) (*p* = 0.003) compared to those without PN. Slopes of fasting glucose, SBP and HDL were not significantly different between those with and without PN. Those with PN had larger AUCs of fasting glucose (103.3 [17.2] vs. 100.1 [15.4]) (*p* < 0.001), SBP (142.8 [12.9] vs. 140.8 [12.8]) (*p* < 0.001), triglycerides (129.2 [54.1] vs. 119.6 [48.9]) (*p* < 0.001), waist circumference (100.0 [12.5] vs. 94.9 [12.5]) (*p* < 0.001) and a smaller AUC of HDL (50.1 [15.0] vs. 54.4 [15.7]) (*p* < 0.001) compared to those without PN.

Those with RN had smaller slopes of triglycerides (1.7 [6.3] vs. 2.5 [6.0]) (*p* = 0.05) and waist circumference (0.6 [0.7] vs. 0.8 [0.8]) (*p* = 0.001) compared to those without RN. Those with RN had larger AUCs of fasting glucose (105.7 [19.9] vs. 101.4 [14.4]) (*p* < 0.001) and SBP (146.1 [13.8] vs. 142.2 [12.9]) (*p* < 0.001) compared to those without RN. Slopes of fasting glucose, SBP, and HDL, and AUCs of HDL, triglyceride, and waist circumference were not different between those with and without RN.

### 3.3 Adjusted Association of Summaries of Longitudinal Metabolic Risk Factors (From Visits 1 to 4) and Microvascular Complications (at Visits 5 or 6)

Larger AUC of fasting glucose (OR: 1.09, 95% CI: 1.05–1.15) and SBP (OR: 1.27, 95% CI: 1.19–1.36) curves associated with greater odds of CKD (Table 2). All metabolic slopes, and AUCs of HDL, triglycerides, and waist circumference were not associated with CKD. Larger AUC of triglyceride (OR: 1.14, 95% CI: 1.04–1.25) and waist circumference (OR: 1.11, 95% CI: 1.07–1.15) were associated with greater odds of PN. Other longitudinal metabolic summaries were not associated with PN. Larger AUC of fasting glucose (OR: 1.13, 95% CI: 1.04–1.22) increased the odds of RN. Other longitudinal metabolic summaries were not associated with RN.

**TABLE 2:** Associations between summaries of longitudinal metabolic risk factors and microvascular complications.

Variables	CKD	PN	RN
Slopes	OR (95% CI)	OR (95% CI)	OR (95% CI)
Fasting glucose (mg/dL)	1.03 (1.00, 1.07)*	0.98 (0.93, 1.02)	0.98 (0.93, 1.03)
SBP (mmHg)	1.06 (1.01, 1.11)*	1.00 (0.95, 1.05)	1.01 (0.94, 1.08)
HDL (mg/dL)	1.00 (0.94, 1.08)	0.94 (0.87, 1.02)	0.98 (0.87, 1.09)
Triglycerides (mg/dL)	0.98 (0.97, 1.00)*	0.99 (0.98, 1.01)	1.00 (0.98, 1.02)
Waist circumference (cm)	1.02 (0.91, 1.14)	0.98 (0.87, 1.10)	0.87 (0.73, 1.03)
AUC			
Fasting glucose (10 mg/dL)	1.09 (1.05, 1.15)*^†^	1.05 (0.99, 1.12)	1.13 (1.04, 1.22)*^†^
SBP (10 mmHg)	1.27 (1.19, 1.36)*^†^	1.04 (0.96, 1.11)	1.11 (1.01, 1.22)*
HDL (10 mg/dL)	0.97 (0.91, 1.04)	1.06 (0.99, 1.14)	1.04 (0.94, 1.15)
Triglycerides (50 mg/dL)	1.06 (0.97, 1.16)	1.14 (1.04, 1.25) *^†^	1.02 (0.89, 1.17)
Waist circumference (5 cm)	1.03 (0.99, 1.07)	1.11 (1.07, 1.15)*^†^	1.00 (0.95, 1.06)

*Note: p* values: *: < 0.05; BH‐adjusted *p* values: ^†^: < 0.05.

Abbreviations: AUC: area under the curve; CKD: chronic kidney disease; HDL: high‐density lipoprotein; PN: peripheral neuropathy; RN: retinopathy; SBP: systolic blood pressure.

### 3.4 Adjusted Associations of Cross‐Sectional Metabolic Risk Factors (at Visit 1) and Microvascular Complications (at Visit 5 or 6)

Larger visit 1 fasting glucose (OR: 1.08, 95% CI: 1.03–1.13), SBP (OR: 1.16, 1.10–1.23), triglycerides (OR: 1.10, 95% CI: 1.02–1.19) and waist circumference (OR: 1.05, 95% CI: 1.01–1.08) associated with greater odds of CKD development, whereas HDL was not (Table S1). Larger triglyceride (OR: 1.13, 95% CI: 1.04–1.23) and waist circumference (OR: 1.12, 95% CI: 1.07–1.16) at visit 1 associated with greater risk of PN. Visit 1 assessments of fasting glucose, SBP and HDL were not associated with PN development. Larger visit 1 assessments of fasting glucose (OR: 1.10, 95% CI: 1.02–1.19) and SBP (OR: 1.15, 95% CI: 1.05–1.25) associated with greater chance of future RN risk. There were no associations between RN and HDL, triglycerides or waist circumference at visit 1.

### 3.5 Comparison of Prediction Metrics From Longitudinal Metabolic Slope and AUC Models (From Visits 1 to 4) to Cross‐Sectional Models (at Visit 1 and Visit 4)

Calibration plots and Hosmer–Lemeshow tests indicated that each model was well‐calibrated (Figure S1A–F). We found that discriminatory capabilities between longitudinal, visit 1, and visit 4 models were very similar for CKD (AUROC range: 0.75–0.76), PN (0.72 for all 3) and RN (AUROC range: 0.65–0.66). The prediction accuracy was measured via Brier scores, all of which were also very similar for CKD (Brier range: 0.14–0.15), PN (0.2 for all 3) and RN (0.15 for all 3). Decision curve analysis revealed the predicted probabilities where clinical decision making would be improved by our logistic regression models. Specifically, our models would improve prediction when the predicted probabilities were between 10% and 70% for CKD, 20% and 75% for PN and 15% and 50% for RN.

For CKD, the model including summaries of longitudinal metabolic risk factors had significantly higher AUROC (0.76, 95% CI: 0.75–0.78) compared to the model with cross‐sectional metabolic risk factor assessments at visit 1 (0.75, 95% CI: 0.74–0.77, *p* value < 0.001) and visit 4 (AUROC: 0.76, 0.74–0.78, *p* value = 0.03) (Table S1). Also, IDI (visit 1: 0.01, 95% CI: 0.01–0.02; visit 4: IDI: 0.01, 95% CI: 0–0.01) and Continuous NRI (visit 1: 0.23, 95% CI: 0.15–0.30; visit 4: NRI: 0.15, 95% CI: 0.08–0.22) indicate the longitudinal summaries models are an improvement in risk discrimination and reclassification over cross‐sectional models. In contrast, inclusion of longitudinal metabolic summaries did not improve AUROC for PN (slope and AUC model: 0.72, 95% CI: 0.70–0.74 vs. visit 1 model 0.72, 95% CI: 0.70–0.74, *p* value = 0.55; visit 4 model: 0.72, 0.70–0.74, *p* value = 0.16) (visit 1 IDI: 0, 95% CI: 0–0; visit 4 IDI: 0, 95% CI: 0–0.01) (visit 1 NRI: 0.05, 95% CI: −0.03–0.13; visit 4 NRI: 0.09, 95% CI: 0.01–0.16) or RN (slope and AUC model: 0.66, 95% CI: 0.63–0.69 vs. visit 1 model 0.66, 95% CI: 0.63–0.69, *p* value = 0.59; visit 4 model: 0.65, 0.62–0.68, *p* value = 0.08) (visit 1 IDI: 0, 95% CI: 0–0; visit 4 IDI: 0, 95% CI: 0–0.01) (visit 1 NRI: −0.04, 95% CI: −0.16–0.07; visit 4 NRI: 0.03, 95% CI: −0.09–0.14) compared to models with solely cross‐sectional metabolic risk factor assessments.

### 3.6 Sensitivity Analyses

In our first sensitivity analysis stratified by diabetes status, we found that SBP AUC was associated with CKD and waist circumference AUC was associated with PN among both those with and without diabetes (Table S3). However, there were some differences among patients that developed versus did not develop diabetes. For CKD, fasting glucose AUC (with diabetes: OR: 1.10, 95% CI: 1.04–1.16; without diabetes: OR: 1.01, 95% CI: 0.87–1.17) was associated with CKD among those with diabetes but not among those without. For PN, triglyceride AUC (with diabetes: OR: 1.05, 95% CI: 0.86–1.27; without diabetes: OR: 1.18, 95% CI: 1.06–1.32) was associated with PN in the group without diabetes but not within the group with diabetes.

The results for the second sensitivity analysis including slopes and baseline values of metabolic risk factors were similar to the slope and AUC models with some subtle differences, primarily regarding metabolic risk factor slopes (Table S4). Specifically, slopes of fasting glucose (primary model: OR: 1.03, 95% CI: 1.00–1.07; sensitivity analysis: OR: 1.07, 95% CI: 1.04–1.11) and SBP (primary model: OR: 1.06, 95% CI: 1.01–1.11; sensitivity analysis: OR: 1.15, 95% CI: 1.09–1.21) were now associated with CKD. In addition, although fasting glucose AUC was associated with RN, baseline fasting glucose was not (primary model (AUC): OR: 1.13, 95% CI: 1.04–1.22; sensitivity analysis (visit 1): OR: 1.10, 95% CI: 1.01–1.19). Finally, baseline SBP was associated with RN, whereas SBP AUC was not (primary model (AUC): OR: 1.11, 95% CI: 1.01–1.22; sensitivity analysis (visit 1): OR: 1.18, 95% CI: 1.07–1.29).

Results from our third sensitivity analysis that included all ARIC participants and imputed missing information using multiple imputations were similar to our primary analysis, with some subtle differences. Compared to our primary analysis, we found that fasting glucose slope (primary model: OR: 1.03, 95% CI: 1.00–1.07; sensitivity analysis: OR: 1.03, 95% CI: 1.01–1.05) and triglyceride AUC (Primary model: OR: 1.06, 95% CI: 0.97–1.16; sensitivity analysis OR: 1.08, 95% CI: 1.03–1.13) were now significantly associated with CKD (Table S5). For PN, triglyceride AUC (primary model: OR: 1.14, 95% CI: 1.04–1.25; sensitivity analysis OR: 1.05, 95% CI: 0.99–1.11) was no longer associated with PN. For RN, the statistical significance of odds ratios was not changed.

The fourth and final sensitivity analysis adjusting for additional confounding variables resulted in similar associations between longitudinal summaries of metabolic risk factors and microvascular complications, with one exception (Table S6). Specifically, triglyceride AUC was no longer associated with PN (primary model: OR: 1.14, 95% CI: 1.04–1.25; sensitivity analysis: OR: 1.10, 95% CI: 0.99–1.22).

### 3.7 Exploratory Analysis: Association Between Summaries of Longitudinal Metabolic Risk Factors (From Visits 1 to 4) and Microvascular Complications (at Visit 5 or 6) by Age Group

#### 3.7.1 Age‐Group Associations Between Summaries of Longitudinal Metabolic Risk Factors (From Visits 1 to 4) and CKD (at Visit 5)

The marginal ORs from the model including interaction terms between longitudinal slopes and AUCs, and age groups showed that the AUC of SBP was associated with CKD in multiple age groups (age 50–54: OR: 1.25, 95% CI: 1.11–1.41; age 55–59: 1.42, 95% CI: 1.25–1.60; age 60–65: 1.33, 95% CI: 1.14–1.54), with the greatest effect size in those aged 55–59 (Figure 2A). Other summaries of longitudinal metabolic risk factors were only associated with CKD in one age group. We found the association between SBP AUC and CKD was significantly larger among those aged 55–59, compared to age 44–49 (*p* value = 0.02).

**FIGURE 2:**
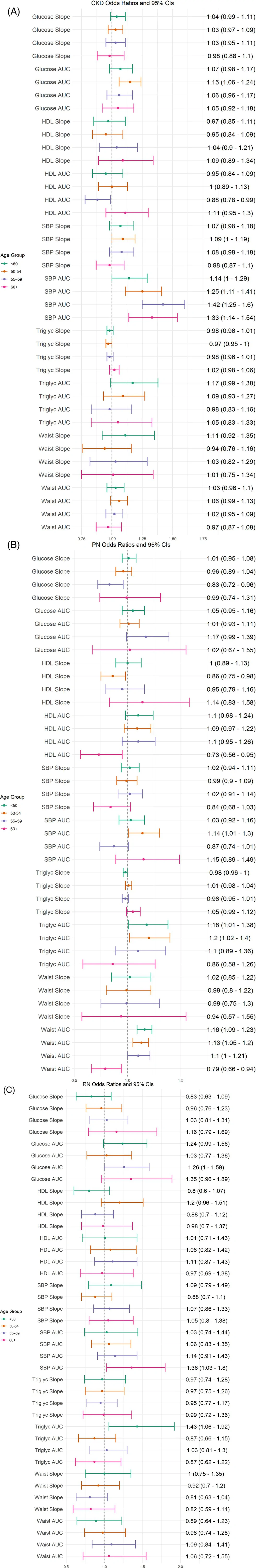
(A) Age‐group analysis of summaries of metabolic risk factors and CKD. (B) Age‐group analysis of summaries of metabolic risk factors and PN. (C) Age‐group analysis of summaries of metabolic risk factors and RN. AUC: area under the curve; CKD: chronic kidney disease; HDL: high‐density lipoprotein; PN: peripheral neuropathy; RN: retinopathy; SBP: systolic blood pressure.

#### 3.7.2 Age‐Group Associations Between Summaries of Longitudinal Metabolic Risk Factors (From Visits 1 to 4) and PN (at Visit 6)

AUC of waist circumference was associated with PN in each age strata (age 44–49: OR: 1.16, 95% CI: 1.09–1.23; age 50–54: 1.13, 95% CI: 1.05–1.20; age 55–59: 1.10, 95% CI: 1.00–1.21; age 60–65: 0.79, 95% CI: 0.66–0.94), with a decreasing effect size with increasing age (Figure 2B). Other summaries of longitudinal metabolic risk factors were only associated with PN in one age group. We found the association between slopes of HDL (*p* value < 0.01) and triglycerides (*p* value = 0.02), and PN were significantly larger, whereas waist circumference association was significantly smaller (*p* value < 0.01), among those aged 60–65 compared to age 44–49. Further, the association between fasting glucose slope and PN was significantly smaller among those aged 55–59 compared to age 44–49.

#### 3.7.3 Age Group Associations Between Summaries of Longitudinal Metabolic Risk Factors (From Visits 1 to 4) and RN (at Visit 5)

There were no clear patterns that emerged through the marginal ORs of the RN interaction model. Specifically, we found only triglyceride AUC (1.43, 95% CI: 1.06–1.92) was associated with RN in those aged 44–49, only fasting glucose AUC (1.26, 95% CI: 1.00–1.59) was associated with RN in ages 55–59, and only SBP AUC (1.36, 95% CI: 1.03–1.80) was associated with RN in the 60–65 age group (Figure 2C). We found associations between HDL slope (*p* value = 0.03) and triglycerides AUC (*p* value = 0.02), and RN were significantly smaller among those aged 50–54, compared to age 44–49. The association of triglycerides AUC and RN was also significantly smaller among those aged 60–65, compared to age 44–49 (*p* value = 0.03).

## 4 Discussion

In a large longitudinal study of persons with and without diabetes, our primary analysis found AUCs, but not slopes of longitudinal metabolic risk factors were associated with future microvascular complications. Specifically, fasting glucose and SBP AUC were associated with CKD, triglycerides and waist circumference AUC with PN, and fasting glucose AUC with RN. However, despite these associations, the longitudinal metabolic summaries did not improve our ability to discern who is likely to have complications compared to a single cross‐sectional assessment (at visit 1 or visit 4), except for a subtle improvement in predicting CKD. Finally, our exploratory analysis indicated that the importance of some longitudinal metabolic profiles changed depending on age at study entry. In particular, the association between SBP AUC for CKD increased with age, whereas the association of waist circumference AUC with PN decreased with age.

We found that AUCs of fasting glucose and SBP were associated with CKD. Further, these associations were consistent, even after adjustment for a comprehensive set of possible confounding factors, such as lifestyle and socioeconomic factors. Further, the results from the second sensitivity analysis assessing associations of slopes and baseline values revealed slopes of fasting glucose and SBP associated with CKD, emphasizing the importance of longitudinal fasting glucose and SBP. Our study adds to the growing body of evidence that longitudinal assessments of SBP and glucose are important for CKD. For fasting glucose, our findings align with prior studies which found that increasing fasting glucose trajectories [7], and elevated but stable [5, 6] fasting glucose trajectories associated with CKD. For SBP, our findings not only align with previous studies that found higher longitudinal trajectories of SBP were associated with CKD outcomes [8, 9, 29], but also indicate the cumulative effect of SBP may also confer risk of future CKD. Given that several past studies found obesity trajectories to be associated with CKD [11, 12, 13], we were surprised to find that waist circumference summaries were not associated with CKD. One explanation is that past studies have not adjusted for other metabolic risk factors such as hypertension or hyperglycaemia. Overall, our findings indicate that adverse longitudinal summaries of hyperglycaemia and hypertension significantly increase the odds of developing CKD.

For PN, we found that a greater AUC of both triglyceride and waist circumference was associated with future PN. To our knowledge, this was the first study to find AUC of triglyceride and waist circumference to be associated with PN. However, this finding is not completely surprising given the substantial evidence linking elevated obesity and triglycerides to PN [30, 31, 32]. In fact, obesity has emerged as the second leading metabolic risk factor for PN [30]. Interestingly, triglyceride AUCs were not associated with PN in our sensitivity analyses that used the full ARIC dataset or after accounting for a comprehensive set of confounding factors. On the other hand, waist circumference AUC remained associated with PN in this sensitivity analysis, highlighting its importance for PN. Our age‐group analysis indicated that waist circumference and triglyceride summaries had the greatest importance in the youngest age group (44–49). This finding highlights the importance that early and sustained obesity and hypertriglyceridemia have for the development of PN. Given that our study only includes adults aged 44 and above, future studies with even younger populations are needed to determine whether the effect of obesity on PN grows in importance among younger populations. To our surprise, both the rate of change and AUC of fasting glucose were not associated with PN. This result is in contrast to the aforementioned Anglo‐Dutch Study of Intensive Treatment in People with Screen Detected Diabetes in Primary Care (ADDITION) [14] and a study of Taiwanese persons with T2D, which both found HbA1c slope to be associated with PN [19]. One possible discrepancy is that both previous studies only included those with diabetes, whereas our cohort included a mixed population with and without diabetes. However, our sensitivity analysis of those with diabetes also found glucose longitudinal summaries were not associated with PN. Taken together, current evidence indicates that AUC of obesity and rate of hyperglycaemia change may be the most important longitudinal metabolic summaries for PN. However, given the limited number of studies and inconsistent evidence, future studies are needed to definitively determine which longitudinal metabolic summaries are associated with future PN risk.

Our results demonstrate that a greater AUC of fasting glucose was associated with RN. This adds to the growing body of evidence that the cumulative effect of having hyperglycaemia increases the risk of RN. In particular, the TODAY [16] study, Kaarniranta et al. [18], Avery et al. [17] and Chang et al. [19] all found that higher sustained trajectories of hyperglycaemia were associated with RN. Despite the robust association between fasting glucose AUC and RN, we did not find that fasting glucose slope was associated with the odds of RN. Overall, current evidence suggests that preventing sustained hyperglycaemia, but not necessarily changing over time, is critical to prevent future RN.

Although multiple summaries of metabolic risk factors from visits 1 to 4 were associated with microvascular complications, we were surprised to find that the discriminatory prediction capability was not consistently improved compared to cross‐sectional models based on metabolic risk factors assessed at visit 1. In fact, longitudinal metabolic summaries only significantly improved the prediction for CKD; however, the improvement to AUROC was only 0.01 and is not likely to be clinically meaningful. One explanation might be that longitudinal metabolic summaries were largely stable during the study. In fact, over the course of 7–11 years, on average fasting glucose only changed by 0.3 to 0.4 mg/dL, SBP by 1.4 mmHg, HDL between −0.1 and −0.2 mg/dL, triglycerides by 2.3 to 2.4 mg/dL, and waist circumference 0.7 to 0.8 cm per year. This stability may be because our study participants were actively enrolled in an epidemiological study with regular and frequent access to care. Thus, it is possible that longitudinal metabolic summaries could be more important in participants in the general population, with poorer metabolic control. Another possibility is that the worsening metabolic profiles occur earlier in the lifespan, and our study only included adults above the age of 43 at visit 1. In contrast, we did find some improvements to prediction capability through NRI for CKD (0.23) compared to visit 1, indicating some modest improvement to CKD classification. Nonetheless, our results indicate slope and AUC models showed statistically significant associations with complications but only limited incremental discrimination and moderate improvement to classification beyond baseline metabolic risk factors in this cohort during midlife, with relatively stable metabolic profiles, and considerable attrition/loss of participants. Future studies are needed to determine whether summaries of metabolic risk factors provide additional information earlier in the lifespan, or in populations with worse or less stable metabolic control, earlier onset diabetes, medication changes or other race/ethnicities.

To our knowledge, our study was the first to comprehensively compare whether the importance of longitudinal metabolic summaries for microvascular complications changes throughout midlife to older adulthood through exploratory analysis. Further, we found that the most important summaries of longitudinal metabolic risk factors for microvascular complications changed throughout the lifespan. For example, for CKD, we found that the importance of AUC of SBP increased with age. This indicates that interventions to address SBP to prevent CKD are likely to be important throughout this period and even more as patients get older. Whereas for PN, we found AUC of waist circumference curve had the greatest association in people aged 44–49, but then decreased with age, and even became protective in those aged 60–65. Therefore, interventions to address obesity to prevent neuropathy are needed earlier and might indicate that weight loss interventions may be less beneficial after the age of 60. Although these findings are similar to the so‐called ‘obesity paradox’ found in conditions such as dementia, where midlife obesity increases risk [33], but late‐life obesity becomes protective, perhaps due to reverse causality [34, 35]. Alternatively, this may be a result of survival bias or unintentional weight loss. Another possibility is that as people age, they become more conscious of health problems, leading them to seek more knowledge and awareness of obesity measures. For example, past studies have found that food preparation, cooking skills and obesity awareness associate with less food cravings, emotional eating and improved metabolic profiles [36, 37]. Overall, our findings suggest that the age at which persons are metabolically impaired differentially impacts whether they will develop microvascular complications, thus providing key implications for metabolic risk factor interventions. However, given the number of statistical comparisons and exploratory nature of this analysis, future, large sample studies are needed to confirm our findings.

Limitations to our study include the incomplete follow‐up assessments for ARIC participants. However, our third sensitivity analysis that included all ARIC participants via multiple imputations yielded similar findings. In addition, our study only included those who were between 44 and 65 years old at visit 1, and thus, we were unable to determine whether longitudinal metabolic summaries earlier or later in the lifespan may have had differential effects on microvascular complications. We also did not have metabolic information between 1999 and 2010, and thus, our analysis was unable to account for metabolic risk factor changes directly prior to our outcome assessments. In addition, given PN and RN were only assessed at the end of follow‐up, we were unable to exclude persons with prevalent PN or RN from our analysis. In addition, we were unable to determine whether participants had a clinical diagnosis of CKD, which would require having eGFR < 60 for at least 3 months or additional evidence of kidney damage. Furthermore, PN was assessed via monofilament testing on the feet, which has poor sensitivity and may miss detecting early or small‐fibre neuropathy [38]. Finally, we defined RN as having mild proliferative diabetic retinopathy, and thus, we did include patients with earlier assessments using ETDRS with a cutoff of 61, which might have missed patients with early stage RN [39]. Finally, ARIC also only included Black and White persons, and thus, it is unclear whether our findings are generalizable to other races or ethnicities.

We found summaries of metabolic risk factors over 9 years during midlife were associated with the risk of developing microvascular complications. We found fasting glucose and SBP AUCs were associated with CKD, triglyceride and waist circumference AUCs with PN, and fasting glucose AUC with RN. However, surprisingly, including these longitudinal metabolic summaries did not improve our ability to determine who is likely to develop these complications, compared to simple cross‐sectional assessments of metabolic risk factors at visits 1 and 4. Our age‐group analysis indicates that the importance of metabolic risk factors for microvascular complications may change across ages 44–65. Future studies are needed to validate our findings and confirm the age‐periods whereby longitudinal metabolic risk factors and their summaries confer the greatest risk for microvascular complications.

## Author Contributions

Cody H. Makinson performed statistical analysis, interpretation of the statistical analysis and wrote the manuscript. Jihyun Park provided an interpretation of the statistical analysis and critical revisions of the manuscript. Eva L. Feldman provided an interpretation of the statistical analysis and critical revisions to the manuscript. Brian C. Callaghan provided an interpretation of the statistical analysis and critical revisions to the manuscript. Evan L. Reynolds was the guarantor, involved in the study design, performed the statistical analysis, interpretation of the statistical analysis and wrote the manuscript.

## Funding

Cody H. Makinson is supported by NIH (R00DK129785). Eva L. Feldman is supported by the United States National Insitutes of Health (R01DK129320, R01DK107956, R01DK130913) and the JDRF (5‐COE‐2019‐861‐S‐B). Brian C. Callaghan is supported by the JDRF. Evan L. Reynolds is supported by NIH (R00DK129785).

## Ethics Statement

This study was approved by the Michigan State University Institutional Review Board.

## Conflicts of Interest

Brian C. Callaghan consults for DynaMed, receives research support from the American Academy of Neurology and performs medical legal consultations including consultations for the Vaccine Injury Compensation Program. The other authors declare no conflicts of interest.

## Supporting information


**Table S1:** Associations between cross‐sectional assessments of metabolic risk factors and microvascular complications.
**Table S2:** Prediction, discrimination and calibration metrics for our longitudinal summaries, visit 1 and visit 4 models.
**Table S3:** Comparison of associations between summaries of longitudinal metabolic risk factors and microvascular complications in those with and without diabetes.
**Table S4:** Logistic regression models determining association between slopes and baseline (visit 1) values of longitudinal metabolic risk factor trajectories and microvascular complications.
**Table S5:** Logistic regression models determining association between slopes and AUCs of longitudinal metabolic risk factor trajectories and microvascular complications using multiple imputations for missing data.
**Table S6:** Logistic regression models determining association between slopes and AUCs of longitudinal metabolic risk factor trajectories and microvascular complications while adjusting for additional exposures.
**Figure S1A:** Calibration plot for CKD main logistic model.
**Figure S1B:** Decision curve for main CKD logistic model.
**Figure S1C:** Calibration plot for PN main logistic model.
**Figure S1D:** Decision curve for main PN logistic model.
**Figure S1E:** Calibration plot for RN main logistic model.
**Figure S1F:** Decision curve for main PN logistic model.

## Data Availability

The data that support the findings of this study are available from Biologic Specimen and Data Repository Information Coordinating Center (BioLINCC). Restrictions apply to the availability of these data, which were used under licence for the current study and therefore are not publicly available. Data are, however, available from the authors upon reasonable request and with permission of NIH BioLINCC.
