# Supplementary material for: Longitudinal Metabolic Risk Factors Are Associated With Microvascular Complications
Source: Diabetes Obes Metab. 2026 Sep 13;28(12):11328–42. doi: 10.1111/dom.71338 (PMC13731042; doi:10.1111/dom.71338)
Supplement: Supplementary file 1 [file DOM-28-11328-s001.docx]

**Supplemental Materials**

**Table S1**: Associations between cross-sectional assessments of metabolic risk factors and microvascular complications

| Variables | | CKD | | PN | RN | |
| --- | --- | --- | --- | --- | --- | --- |
| Visit 1 | **OR (95% CI)** | | **OR (95% CI)** | | | **OR (95% CI)** |
| Fasting Glucose (10 mg/dL) | 1.08 (1.03,1.13)*† | | 1.04 (0.98,1.09) | | | 1.10 (1.02,1.19)*† |
| SBP (10 mmHg) | 1.16 (1.10,1.23)*† | | 1.00 (0.93,1.06) | | | 1.15 (1.05,1.25)*† |
| HDL (10 mmol/L) | 0.98 (0.92,1.04) | | 1.06 (0.99,1.13) | | | 1.04 (0.94,1.14) |
| Triglycerides (50 mmol/L) | 1.10 (1.02,1.19)*† | | 1.13 (1.04,1.23)*† | | | 1.05 (0.93,1.18) |
| Waist Circumference (5 cm) | 1.05 (1.01,1.08)*† | | 1.12 (1.07,1.16)*† | | | 1.00 (0.95,1.06) |

Table S1 Legend: Legend: CKD: chronic kidney disease; PN: peripheral neuropathy; RN: retinopathy; HDL: high-density lipoprotein; SBP: systolic blood pressure; AUC: area under the curve; p – values: *: <0.05; BH-adjusted p-values: †: <0.05

**Table S2**: Prediction, Discrimination, and Calibration metrics for our Longitudinal Summaries, visit 1, and visit 4 models

|  | AUC + Slope Model | Visit 1 Model | Visit 4 Model |
| --- | --- | --- | --- |
| **CKD** | | | |
| AUROC | 0.764 | 0.754 | 0.759 |
| Sensitivity | 0.228 | 0.213 | 0.222 |
| Specificity | 0.961 | 0.962 | 0.961 |
| NPV | 0.815 | 0.812 | 0.814 |
| PPV | 0.622 | 0.610 | 0.614 |
| Brier | 0.143 | 0.145 | 0.144 |
| HL Test | P=0.412 | P=0.800 | P=0.574 |
| IDI |  | 0.013 (p<0.01) | 0.006 (p<0.01) |
| NRI |  | 0.225 (p<0.01) | 0.151 (p<0.01) |
| DeLong’s Test |  | 0.01 (p<0.01) | 0.005 (p=0.034) |
| **PN** | | | |
| AUROC | 0.723 | 0.722 | 0.719 |
| Sensitivity | 0.414 | 0.425 | 0.413 |
| Specificity | 0.853 | 0.849 | 0.860 |
| NPV | 0.711 | 0.714 | 0.713 |
| PPV | 0.625 | 0.624 | 0.636 |
| Brier | 0.200 | 0.200 | 0.201 |
| HL Test | P=0.801 | P=0.895 | P=0.650 |
| IDI |  | 0.002 (p=0.181) | 0.004 (p=0.007) |
| NRI |  | 0.049 (p=0.210) | 0.087 (p=0.027) |
| DeLong’s Test |  | 0.001 (p=0.552) | 0.004 (p=0.160) |
| **RN** | | | |
| AUROC | 0.658 | 0.661 | 0.648 |
| Sensitivity | 0.020 | 0.017 | 0.022 |
| Specificity | 0.998 | 0.996 | 0.999 |
| NPV | 0.804 | 0.803 | 0.804 |
| PPV | 0.700 | 0.500 | 0.889 |
| Brier | 0.150 | 0.150 | 0.151 |
| HL Test | P=0.659 | P=0.968 | P=0.085 |
| IDI |  | 0 (p=0.912) | 0.004 (p=0.066) |
| NRI |  | -0.041 (p=0.484) | 0.026 (p=0.666) |
| DeLong’s Test |  | -0.003 (p=0.587) | 0.01 (p=0.084) |

Table S2 Legend: CKD: chronic kidney disease; PN: peripheral neuropathy; RN: retinopathy; HDL: high-density lipoprotein; SBP: systolic blood pressure; AUC: area under the curve; AUROC: area under receiver operator characteristic curve; NPV: negative predictive value, PPV: positive predictive value, HL: Hosmer-Lemeshow, IDI: integrated discriminant improvement; NRI: net reclassification improvement

**Table S3**: Comparison of associations between summaries of longitudinal metabolic risk factors and microvascular complications in those with and without diabetes

| Variables | CKD with  Diabetes  N=918 | CKD without  N=3,286 | | | PN with Diabetes  N=502 | PN  Without  N=2,286 | | RN with Diabetes  N=415 | RN Without  N=1,377 |
| --- | --- | --- | --- | --- | --- | --- | --- | --- | --- |
| Slopes | OR (95% CI) | | OR (95% CI) | OR (95% CI) | | OR (95% CI) | OR (95% CI) | | OR (95% CI) |
| Fasting Glucose (mg/dL) | 1.03 (1.00,1.07) | | 0.96 (0.85,1.07) | 0.97 (0.92,1.02) | | 0.98  (0.87,1.09) | 0.98 (0.92,1.04) | | 0.94 (0.80,1.11) |
| SBP (mmHg) | 1.03 (0.94,1.13) | | 1.08 (1.02,1.14)  * | 0.98 (0.88,1.10) | | 1.00  (0.95,1.07) | 1.06 (0.93,1.21) | | 0.99 (0.91,1.08) |
| HDL (mg/dL) | 1.06 (0.92,1.23) | | 0.98 (0.90,1.07) | 0.94 (0.79,1.11) | | 0.94  (0.86,1.03) | 0.80 (0.63,1.02) | | 1.04 (0.91,1.18) |
| Triglycerides (mg/dL) | 0.98 (0.96,1.00) | | 0.99 (0.97,1.01) | 0.99 (0.96,1.02) | | 0.99  (0.98,1.01) | 0.99 (0.95,1.03) | | 1.00 (0.98,1.03) |
| Waist Circumference (cm) | 1.10 (0.89,1.36) | | 0.99 (0.87,1.13) | 1.02 (0.80,1.31) | | 0.96  (0.84,1.11) | 0.85 (0.60,1.20) | | 0.88 (0.71,1.03) |
| AUC |  | |  |  | |  |  | |  |
| Fasting Glucose (10 mg/dL) | 1.10 (1.04,1.16)  *† | | 1.01 (0.87,1.17) | 1.06 (0.99,1.14) | | 0.98  (0.85,1.14) | 1.16 (1.04,1.28) * | | 1.08 (0.87,1.33) |
| SBP (10 mmHg) | 1.32 (1.16,1.50)  *† | | 1.26 (1.17,1.36)  *† | 1.00 (0.85,1.18) | | 1.05  (0.96,1.13) | 1.22 (1.01,1.49)* | | 1.10 (0.97,1.23) |
| HDL (10 mg/dL) | 1.06 (0.92,1.24) | | 0.94 (0.87,1.02) | 1.16 (0.97,1.39) | | 1.04  (0.96,1.13) | 1.05 (0.83,1.32) | | 1.04 (0.92,1.16) |
| Triglycerides (50 mg/dL) | 1.17 (1.01,1.36) * | | 1.01 (0.90,1.13) | 1.05 (0.86,1.27) | | 1.18  (1.06,1.32)  *† | 1.20 (0.94,1.53) | | 0.97 (0.82,1.14) |
| Waist Circumference (5 cm) | 1.05 (0.99,1.12) | | 1.03 (0.98,1.08) | 1.12 (1.04,1.21)  *† | | 1.10  (1.06,1.16)  *† | 1.04 (0.94,1.15) | | 0.99 (0.92,1.07) |

Table S3 Legend: CKD: chronic kidney disease; PN: peripheral neuropathy; RN: retinopathy; HDL: high-density lipoprotein; SBP: systolic blood pressure; AUC: area under the curve; p – values: *: <0.05; BH-adjusted p-values: †: <0.05

**Table S4**: Logistic regression models determining association between slopes and baseline (visit 1) values of longitudinal metabolic risk factor trajectories and microvascular complications

| Variables | CKD  AUROC=0.74 | | PN  AUROC=0.72 | | RN  AUROC=0.66 | |
| --- | --- | --- | --- | --- | --- | --- |
| Slopes | | OR (95% CI) | | OR (95% CI) | | OR (95% CI) |
| Fasting Glucose (mg/dL) | | 1.07 (1.04,1.11)*† | | 1.00 (0.95,1.04) | | 1.02 (0.97,1.07) |
| SBP (mmHg) | | 1.15 (1.09,1.21)*† | | 1.00 (0.95,1.06) | | 1.07 (0.99, 1.15) |
| HDL (mg/dL) | | 1.00 (0.93,1.08) | | 0.96 (0.89,1.04) | | 0.99 (0.88,1.12) |
| Triglycerides (mg/dL) | | 0.99 (0.98,1.01) | | 1.00 (0.99,1.02) | | 1.00 (0.98,1.02) |
| Waist Circumference (cm) | | 1.04 (0.094,1.16) | | 1.07 (0.95,1.20) | | 0.87 (0.73,1.03) |
| Baseline | |  | |  | |  |
| Fasting Glucose  (10 mg/dL) | | 1.09 (1.05,1.15)*† | | 1.04 (0.98,1.10) | | 1.10 (1.01,1.19)* |
| SBP (10 mmHg) | | 1.24 (1.16,1.32)*† | | 1.00 (0.93,1.07) | | 1.18 (1.07,1.29)*† |
| HDL (10 mg/dL) | | 0.98 (0.92,1.05) | | 1.05 (0.98,1.13) | | 1.03 (0.93,1.15) |
| Triglycerides  (50 mg/dL) | | 1.08 (0.99,1.17) | | 1.14 (1.04,1.24)*† | | 1.05 (0.92,1.18) |
| Waist Circumference  (5 cm) | | 1.03 (1.00,1.07) | | 1.12 (1.07,1.16)*† | | 1.00 (0.94,1.05) |

Table S4 Legend: CKD: chronic kidney disease; PN: peripheral neuropathy; RN: retinopathy; HDL: high-density lipoprotein; SBP: systolic blood pressure; AUC: area under the curve; p – values: *: <0.05; BH-adjusted p-values: †: <0.05

**Table S5:** Logistic regression models determining association between slopes and AUCs of longitudinal metabolic risk factor trajectories and microvascular complications using multiple imputations for missing data

| Variables | CKD | | PN | | RN | |
| --- | --- | --- | --- | --- | --- | --- |
| Slopes | | OR (95% CI) | | OR (95% CI) | | OR (95% CI) |
| Fasting Glucose (mg/dL) | | 1.03 (1.01, 1.05)*† | | 0.98 (0.95,1.01) | | 0.99 (0.95, 1.03) |
| SBP (mmHg) | | 1.03 (1.00, 1.07) | | 0.99 (0.94, 1.04) | | 1.02 (0.96, 1.08) |
| HDL (mg/dL) | | 1.00 (0.95, 1.06) | | 0.97, 0.91, 1.04) | | 1.00 (0.91, 1.10) |
| Triglycerides (mg/dL) | | 1.00 (0.99, 1.01) | | 1.00 (0.99, 1.01) | | 1.00 (0.99, 1.02) |
| Waist Circumference (cm) | | 0.93 (0.86, 1.01) | | 0.98 (0.89, 1.09) | | 0.89 (0.77, 1.03) |
| AUC | |  | |  | |  |
| Fasting Glucose  (10 mg/dL) | | 1.07 (1.04, 1.09)*† | | 1.04 (1.00, 1.08)* | | 1.09 (1.04, 1.15)*† |
| SBP (10 mmHg) | | 1.21 (1.15, 1.26)*† | | 1.02 (0.96, 1.09) | | 1.07 (0.99, 1.15) |
| HDL (10 mg/dL) | | 0.96 (0.91, 1.01) | | 1.01 (0.95, 1.08) | | 1.03 (0.95, 1.12) |
| Triglycerides  (50 mg/dL) | | 1.08 (1.03, 1.13)*† | | 1.05 (0.99, 1.11) | | 0.97 (0.89,1.06) |
| Waist Circumference  (5 cm) | | 1.02 (0.99, 1.05) | | 1.09 (1.05, 1.12)*† | | 1.02 (0.97, 1.06) |

Table S5 Legend: CKD: chronic kidney disease; PN: peripheral neuropathy; RN: retinopathy; HDL: high-density lipoprotein; SBP: systolic blood pressure; AUC: area under the curve; p – values: *: <0.05; BH-adjusted p-values: †: <0.05

**Table S6**: Logistic regression models determining association between slopes and AUCs of longitudinal metabolic risk factor trajectories and microvascular complications while adjusting for additional exposures

| Variables | CKD | | PN | | RN | |
| --- | --- | --- | --- | --- | --- | --- |
| Slopes | | OR (95% CI) | | OR (95% CI) | | OR (95% CI) |
| Fasting Glucose (mg/dL) | | 1.03 (0.99, 1.07) | | 0.97 (0.93, 1.02) | | 0.98 (0.92, 1.03) |
| SBP (mmHg) | | 1.06 (1.01,1.12)* | | 1.00 (0.95, 1.07) | | 1.03 (0.95, 1.11) |
| HDL (mg/dL) | | 0.98 (0.91, 1.06) | | 0.96 (0.88,1.04) | | 1.02 (0.90, 1.15) |
| Triglycerides (mg/dL) | | 0.98 (0.97, 1.00)* | | 0.99 (0.98, 1.01) | | 1.00 (0.98, 1.03) |
| Waist Circumference (cm) | | 1.01 (0.89, 1.14) | | 0.99 (0.86, 1.12) | | 0.83 (0.68, 1.00) |
| AUC | |  | |  | |  |
| Fasting Glucose  (10 mg/dL) | | 1.10 (1.04, 1.16)*† | | 1.08 (1.01, 1.15)* | | 1.14 (1.05,1.23)*† |
| SBP (10 mmHg) | | 1.27 (1.19, 1.37)*† | | 1.03 (0.95, 1.12) | | 1.08 (0.96,1.20) |
| HDL (10 mg/dL) | | 0.99 (0.92, 1.07) | | 1.04 (0.96, 1.13) | | 1.04 (0.93, 1.17) |
| Triglycerides  (50 mg/dL) | | 1.06 (0.97, 1.17) | | 1.10 (0.99, 1.22) | | 1.02 (0.88, 1.17) |
| Waist Circumference  (5 cm) | | 1.02 (0.98, 1.06) | | 1.11 (1.06, 1.16)*† | | 0.98 (0.92, 1.04) |

Table S6 Legend: CKD: chronic kidney disease; PN: peripheral neuropathy; RN: retinopathy; HDL: high-density lipoprotein; SBP: systolic blood pressure; AUC: area under the curve; p – values: *: <0.05; BH-adjusted p-values: †: <0.05

**Supplemental Figure 1A**: Calibration Plot for CKD main Logistic Model

Supplemental Figure 1A Legend: CKD: chronic kidney disease

**Supplemental Figure 1B**: Decision Curve for main CKD Logistic Model

Supplemental Figure 1B Legend: CKD: chronic kidney disease

**Supplemental Figure 1C**: Calibration Plot for PN main Logistic Model

Supplemental Figure 1C Legend: PN: peripheral neuropathy

**Supplemental Figure 1D**: Decision Curve for main PN Logistic Model

Supplemental Figure 1D Legend: PN: peripheral neuropathy

**Supplemental Figure 1E**: Calibration Plot for RN main Logistic Model

Supplemental Figure 1E Legend: RN: retinopathy

**Supplemental Figure 1F**: Decision Curve for main PN Logistic Model

Supplemental Figure 1F Legend: RN: retinopathy
